# Supplementary material for: Postoperative complications and hospital costs following small bowel resection surgery
Source: PLoS One. 2020 Oct 21;15(10):e0241020. doi: 10.1371/journal.pone.0241020 (PMC7577438; doi:10.1371/journal.pone.0241020)
Supplement: S1 Appendix — (DOCX) [file pone.0241020.s001.docx]

S1 Fig. Correlation analysis results between complications, total hospital cost, and other collected variables. Data presented as corresponding correlation coefficient and 95% confidence intervals. *: P<0.016 for complication, number of complications, and Clavien-Dindo classification, P<0.050 for grand total.


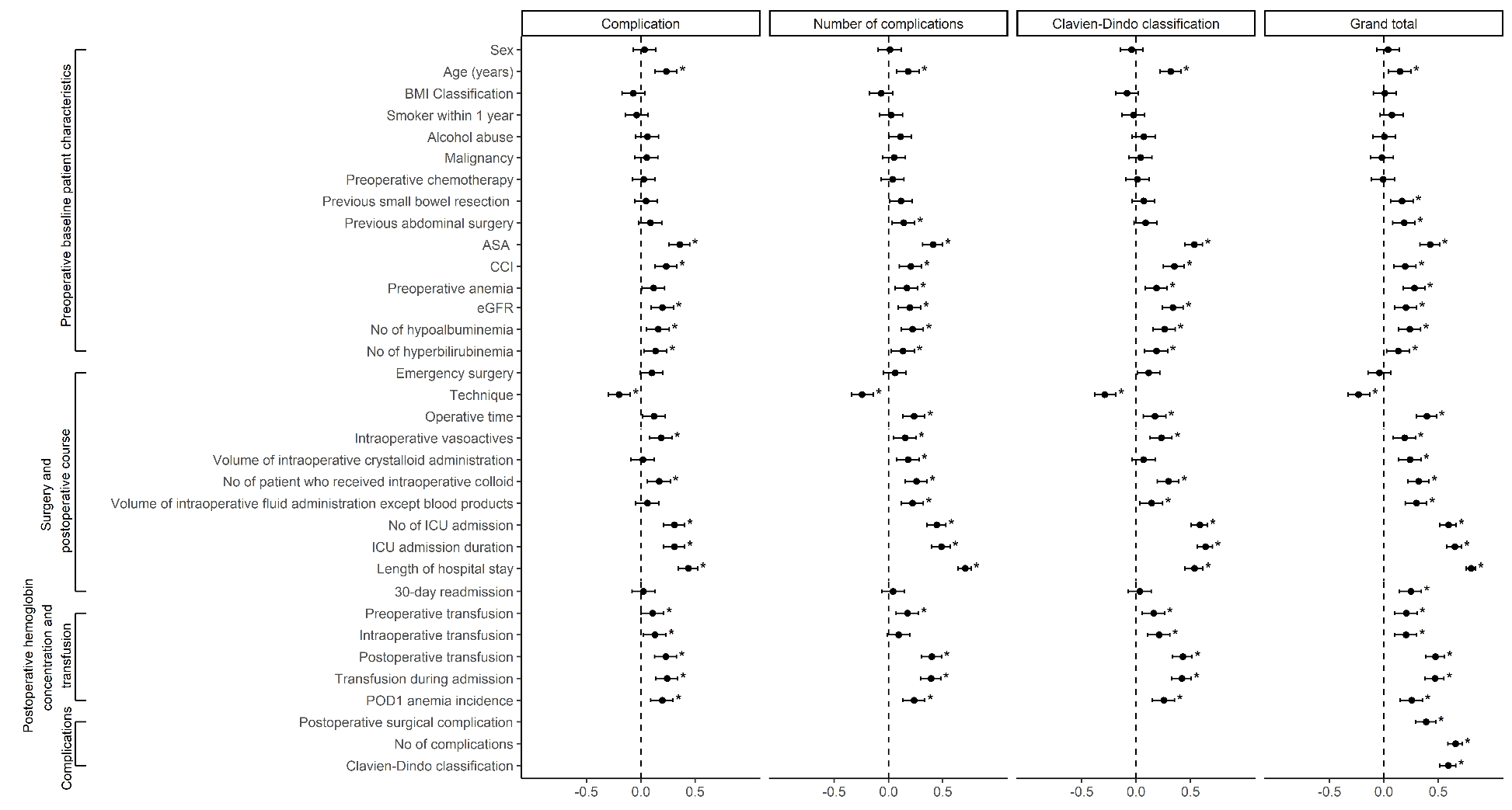


.

S2 Fig. Unadjusted Median cost according to the number of complications.


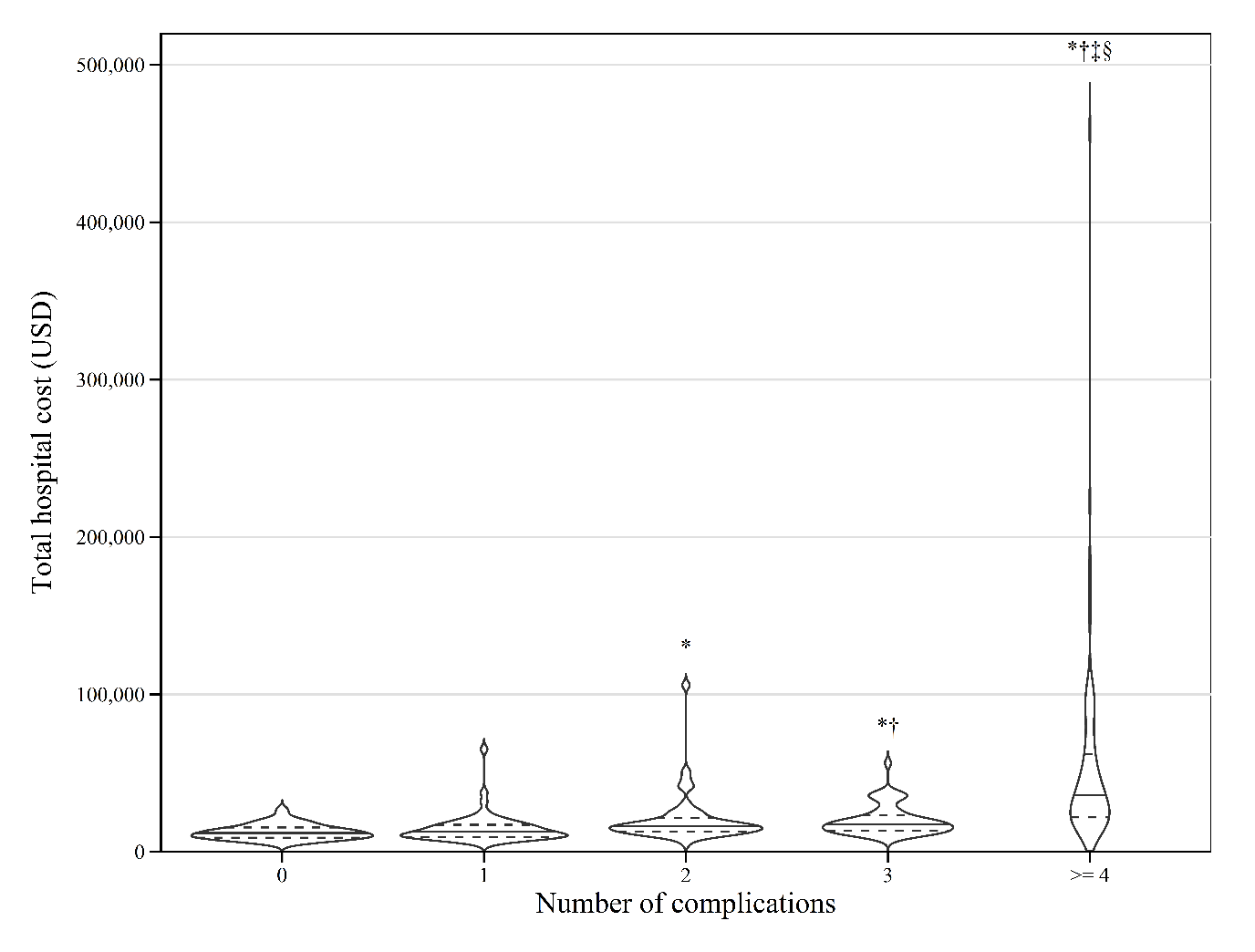


The median cost is increased significantly according to the number of complications (Kruskal-Wallis test, P<0.001, $\eta_{H}^{2}$ = 0.45). *: P<0.010 vs. no complication, †: P<0.010 vs. one complication, ‡: P<0.010 vs. two complications, §: P<0.010 vs. three complications.

S3 Fig. Unadjusted median hospital cost according to the Clavien-Dindo grading of surgical complications.


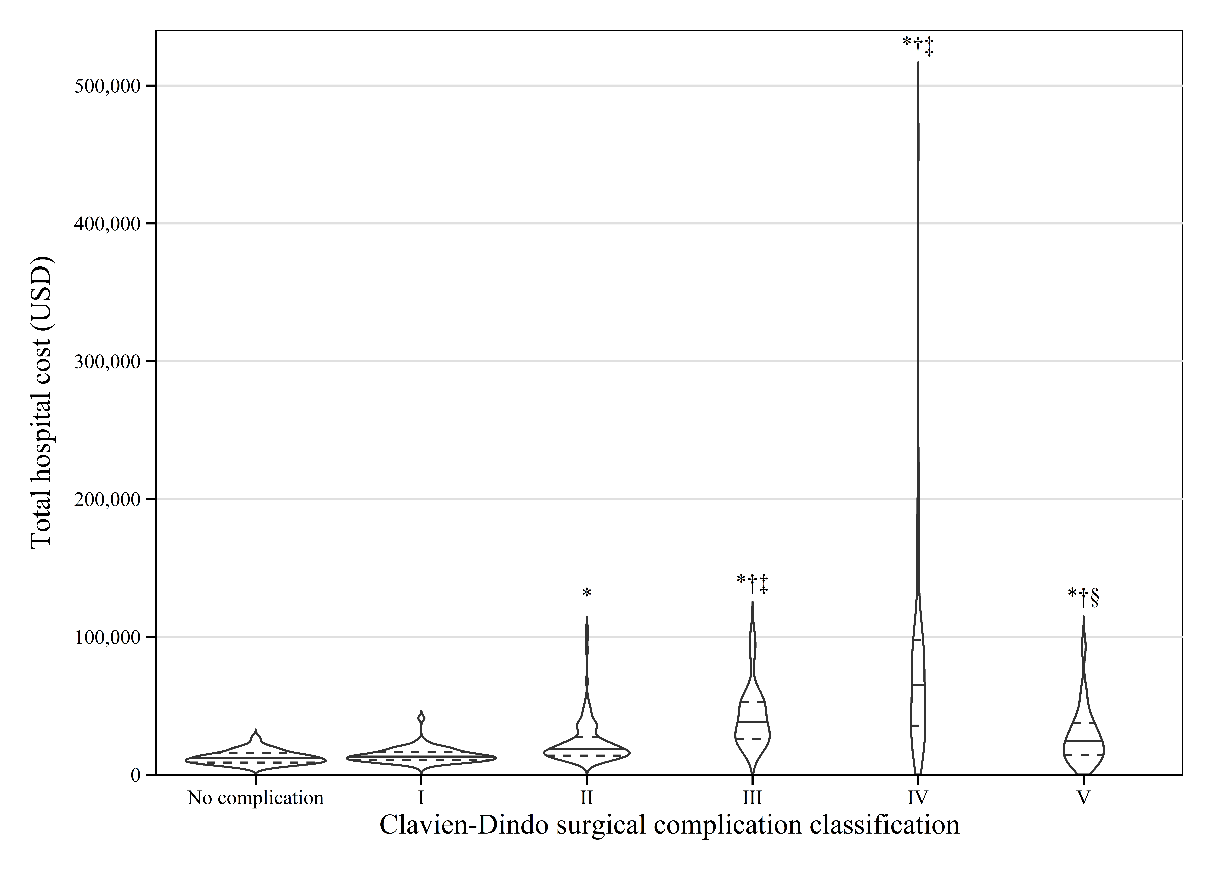


The median cost is increased significantly according to the number of complications (Kruskal-Wallis test, P<0.001, $\eta_{H}^{2}$ = 0.42). *: P<0.0083 vs. CVD grade 0, †: P<0.0083 vs. CVD grade I, ‡: P<0.0083 vs. CVD grade II, §: P<0.0083 vs. CVD grade IV.

S1 Table. International Statistical Classification of Diseases and Related Health Problems 10th Revision (ICD-10) Codes specific to small bowel resections.

| ICD code |
| --- |
| 30565-00 Resection of small intestine with formation of stoma |
| 30515-03 Ileocolic resection with anastomosis |
| 30515-04 Laparoscopic ileocolic resection with anastomosis |
| 30515-06 Laparoscopic ileocolic resection with formation of stoma |
| 30566-00 Resection of small intestine with anastomosis |
| 30515-05 Ileocolic resection with formation of stoma |

S2 Table. Type and severity of complications.

| **Type of complication** | **Grade I** | | **Grade II** | | **Grade III** | | **Grade IV** | | **Grade V** | | **Total** |
| --- | --- | --- | --- | --- | --- | --- | --- | --- | --- | --- | --- |
|  | **n** | **%** | **n** | **%** | **n** | **%** | **n** | **%** | **n** | **%** | **n** |
|  |  |  |  |  |  |  |  |  |  |  |  |
| **Cardiovascular** | 96 | 46.2% | 56 | 26.9% | 4 | 1.9% | 38 | 18.3% | 14 | 6.7% | 208 |
| Myocardial infarction | 2 | 40.0% | 1 | 20.0% | 1 | 20.0% | 1 | 20.0% | 0 | 0.0% | 5 |
| Congestive cardiac failure/fluid overload | 23 | 92.0% | 2 | 8.0% | 0 | 0.0% | 0 | 0.0% | 0 | 0.0% | 25 |
| Hypotension (volume depletion or vasoplegia) requiring treatment | 31 | 35.6% | 5 | 5.7% | 0 | 0.0% | 37 | 42.5% | 14 | 16.1% | 87 |
| Hypertension requiring treatment | 1 | 6.7% | 14 | 93.3% | 0 | 0.0% | 0 | 0.0% | 0 | 0.0% | 15 |
| Arrythmia (atrial fibrillation or flutter) | 3 | 8.8% | 30 | 88.2% | 1 | 2.9% | 0 | 0.0% | 0 | 0.0% | 34 |
| Brady/tachycardia requiring urgent clinic review | 29 | 87.9% | 4 | 12.1% | 0 | 0.0% | 0 | 0.0% | 0 | 0.0% | 33 |
| Other cardiac | 7 | 77.8% | 0 | 0.0% | 2 | 22.2% | 0 | 0.0% | 0 | 0.0% | 9 |
|  |  |  |  |  |  |  |  |  |  |  |  |
| **Pulmonary** | 31 | 36.5% | 36 | 42.4% | 4 | 4.7% | 11 | 12.9% | 3 | 3.5% | 85 |
| Pneumonia | 0 | 0.0% | 25 | 100.0% | 0 | 0.0% | 0 | 0.0% | 0 | 0.0% | 25 |
| Pulmonary congestion/pleural effusion | 8 | 57.1% | 2 | 14.3% | 4 | 28.6% | 0 | 0.0% | 0 | 0.0% | 14 |
| Pulmonary embolus | 0 | 0.0% | 2 | 66.7% | 0 | 0.0% | 1 | 33.3% | 0 | 0.0% | 3 |
| Respiratory failure/atelectasis | 13 | 41.9% | 5 | 16.1% | 0 | 0.0% | 10 | 32.3% | 3 | 9.7% | 31 |
| Other pulmonary | 10 | 83.3% | 2 | 16.7% | 0 | 0.0% | 0 | 0.0% | 0 | 0.0% | 12 |
|  |  |  |  |  |  |  |  |  |  |  |  |
| **Gastrointestinal** | 73 | 33.3% | 98 | 44.7% | 46 | 21.0% | 0 | 0.0% | 2 | 0.9% | 219 |
| Anastomotic leak | 0 | 0.0% | 0 | 0.0% | 16 | 94.1% | 0 | 0.0% | 1 | 5.9% | 17 |
| Ileus | 8 | 17.8% | 33 | 73.3% | 4 | 8.9% | 0 | 0.0% | 0 | 0.0% | 45 |
| Intra-abdominal collection | 0 | 0.0% | 6 | 33.3% | 12 | 66.7% | 0 | 0.0% | 0 | 0.0% | 18 |
| Surgical site infection | 8 | 18.6% | 30 | 69.8% | 5 | 11.6% | 0 | 0.0% | 0 | 0.0% | 43 |
| Total parenteral nutrition | 0 | 0.0% | 66 | 100.0% | 0 | 0.0% | 0 | 0.0% | 0 | 0.0% | 66 |
| High stomal output | 0 | 0.0% | 9 | 100.0% | 0 | 0.0% | 0 | 0.0% | 0 | 0.0% | 9 |
| Nausea and vomiting | 15 | 93.8% | 1 | 6.3% | 0 | 0.0% | 0 | 0.0% | 0 | 0.0% | 16 |
| Diarrhoea | 12 | 63.2% | 7 | 36.8% | 0 | 0.0% | 0 | 0.0% | 0 | 0.0% | 19 |
| Constipation | 16 | 94.1% | 1 | 5.9% | 0 | 0.0% | 0 | 0.0% | 0 | 0.0% | 17 |
| Gastrointestinal bleed | 2 | 14.3% | 7 | 50.0% | 5 | 35.7% | 0 | 0.0% | 0 | 0.0% | 14 |
| Dysphagia/reflux | 5 | 100.0% | 0 | 0.0% | 0 | 0.0% | 0 | 0.0% | 0 | 0.0% | 5 |
| Ischemic bowel/necrotic stoma | 0 | 0.0% | 0 | 0.0% | 3 | 75.0% | 0 | 0.0% | 1 | 25.0% | 4 |
| Other gastrointestinal | 7 | 58.3% | 4 | 33.3% | 1 | 8.3% | 0 | 0.0% | 0 | 0.0% | 12 |
|  |  |  |  |  |  |  |  |  |  |  |  |
| **Hematological** | 17 | 21.5% | 60 | 75.9% | 2 | 2.5% | 0 | 0.0% | 0 | 0.0% | 79 |
| Post-operative blood transfusion | 0 | 0.0% | 66 | 100.0% | 0 | 0.0% | 0 | 0.0% | 0 | 0.0% | 66 |
| Postoperative anemia | 7 | 12.3% | 50 | 87.7% | 0 | 0.0% | 0 | 0.0% | 0 | 0.0% | 57 |
| Febrile neutropenia | 0 | 0.0% | 1 | 100.0% | 0 | 0.0% | 0 | 0.0% | 0 | 0.0% | 1 |
| Thrombosis | 1 | 10.0% | 8 | 80.0% | 1 | 10.0% | 0 | 0.0% | 0 | 0.0% | 10 |
| Other hematological | 9 | 81.8% | 1 | 9.1% | 1 | 9.1% | 0 | 0.0% | 0 | 0.0% | 11 |
|  |  |  |  |  |  |  |  |  |  |  |  |
| **Renal** | 39 | 52.7% | 18 | 24.3% | 2 | 2.7% | 14 | 18.9% | 1 | 1.4% | 74 |
| Acute kidney injury | 24 | 60.0% | 1 | 2.5% | 0 | 0.0% | 14 | 35.0% | 1 | 2.5% | 40 |
| Urinary tract infection | 1 | 7.1% | 13 | 92.9% | 0 | 0.0% | 0 | 0.0% | 0 | 0.0% | 14 |
| Urinary retention | 6 | 50.0% | 4 | 33.3% | 2 | 16.7% | 0 | 0.0% | 0 | 0.0% | 12 |
| Hematuria | 1 | 100.0% | 0 | 0.0% | 0 | 0.0% | 0 | 0.0% | 0 | 0.0% | 1 |
| Other renal | 7 | 100.0% | 0 | 0.0% | 0 | 0.0% | 0 | 0.0% | 0 | 0.0% | 7 |
|  |  |  |  |  |  |  |  |  |  |  |  |
| **Metabolic** | 117 | 63.6% | 64 | 34.8% | 2 | 1.1% | 1 | 0.5% | 0 | 0.0% | 184 |
| Electrolyte imbalance | 96 | 97.0% | 3 | 3.0% | 0 | 0.0% | 0 | 0.0% | 0 | 0.0% | 99 |
| Metabolic acidosis | 3 | 50.0% | 3 | 50.0% | 0 | 0.0% | 0 | 0.0% | 0 | 0.0% | 6 |
| Other metabolic | 18 | 22.8% | 58 | 73.4% | 2 | 2.5% | 1 | 1.3% | 0 | 0.0% | 79 |
|  |  |  |  |  |  |  |  |  |  |  |  |
| **Dermatological** | 7 | 63.6% | 4 | 36.4% | 0 | 0.0% | 0 | 0.0% | 0 | 0.0% | 11 |
| Pressure sore | 3 | 100.0% | 0 | 0.0% | 0 | 0.0% | 0 | 0.0% | 0 | 0.0% | 3 |
| Skin rash/allergy | 2 | 40.0% | 3 | 60.0% | 0 | 0.0% | 0 | 0.0% | 0 | 0.0% | 5 |
| Other dermatological | 2 | 66.7% | 1 | 33.3% | 0 | 0.0% | 0 | 0.0% | 0 | 0.0% | 3 |
|  |  |  |  |  |  |  |  |  |  |  |  |
| **Neurological** | 16 | 44.4% | 19 | 52.8% | 0 | 0.0% | 0 | 0.0% | 1 | 2.8% | 36 |
| Delirium | 12 | 40.0% | 18 | 60.0% | 0 | 0.0% | 0 | 0.0% | 0 | 0.0% | 30 |
| Postoperative stroke | 0 | 0.0% | 0 | 0.0% | 0 | 0.0% | 0 | 0.0% | 1 | 100.0% | 1 |
| Other neurological | 4 | 80.0% | 1 | 20.0% | 0 | 0.0% | 0 | 0.0% | 0 | 0.0% | 5 |
|  |  |  |  |  |  |  |  |  |  |  |  |
| **Other** | 79 | 49.7% | 72 | 45.3% | 4 | 2.5% | 2 | 1.3% | 2 | 1.3% | 159 |
| Uncontrolled postoperative pain/opioid side effect | 26 | 60.5% | 16 | 37.2% | 1 | 2.3% | 0 | 0.0% | 0 | 0.0% | 43 |
| Alcohol withdrawal | 0 | 0.0% | 2 | 100.0% | 0 | 0.0% | 0 | 0.0% | 0 | 0.0% | 2 |
| Mechanical fall | 2 | 100.0% | 0 | 0.0% | 0 | 0.0% | 0 | 0.0% | 0 | 0.0% | 2 |
| Intractable hiccups | 0 | 0.0% | 2 | 100.0% | 0 | 0.0% | 0 | 0.0% | 0 | 0.0% | 2 |
|  |  |  |  |  |  |  |  |  |  |  |  |
| **Total** | 475 | 45.0% | 427 | 40.5% | 64 | 6.1% | 66 | 6.3% | 23 | 2.2% | 1055 |

| S3 Table. Presence of any complication and hospital costs adjusted for predefined variables. Estimated median and 95% confidence intervals of variables at 25th, 50th, and 75th percentiles of hospital cost. | | | | | | | | |
| --- | --- | --- | --- | --- | --- | --- | --- | --- |
| **Variables** | **Number (proportion)** | **25th percentile** | | **50th percentile** | | **75th percentile** | |  |
|  |  | **Median (95%CI)** | **P value** | **Median**  **(95%CI)** | **P value** | **Median (95%CI)** | **P value** |  |
| **Presence of any complication** | | | | | | | |  |
| No | 64 (18.4) | (Reference) |  | (Reference) |  | (Reference) |  |  |
| Yes | 284 (81.6) | 3,038.04 (482.08 – 5,594.00) | 0.021 | 4,187.10 (1,264.89 – 7,109.31) | 0.005* | 5,795.82 (965.65 – 10,625.99) | 0.020 |  |
| **CCI** | NA | 28.53 (-476.80 – 533.86) | 0.912 | 253.04 (-274.09 – 780.18) | 0.348 | 275.25 (-938.49 – 1489.00) | 0.657 |  |
| **Anemia** | | | | | | | |  |
| No | 208 (59.8) | (Reference) |  | (Reference) |  | (Reference) |  |  |
| Yes | 140 (40.2) | 1,405.5 (-1,098.63 – 3,909.62) | 0.272 | 2,419.73 (-418.86 – 5,258.33) | 0.096 | 3,981.05 (-1,404.84 – 9,366.94) | 0.149 |  |
| **Emergency surgery** | | | | | | | |  |
| No | 97 (27.9) | (Reference) |  | (Reference) |  | (Reference) |  |  |
| Yes | 251 (72.1) | -1,382.38 (-4,122.65 – 1,357.90) | 0.324 | -2,336.96 (-5,092.30 – 418.38) | 0.098 | -3,132.67 (-8,694.39 – 2,429.05) | 0.271 |  |
| **Technique** | | | | | | | |  |
| Laparoscopy | 61 (17.5) | (Reference) |  | (Reference) |  | (Reference) |  |  |
| Laparotomy | 287 (82.5) | 861.71 (-1,635.91 – 3,359.34) | 0.500 | 2,847.20 (107.81 – 5,586.60) | 0.043 | 7,026.82 (1,933.26 – 1,2120.37) | 0.007* |  |
| **Intraoperative fluid** | NA | 1,984.44 (733.10 – 3,235.78) | 0.002* | 3,026.44 (1,400.38 – 4,652.51) | <0.001* | 1,708.33 (-529.79 – 3,946.44) | 0.136 |  |
| **Transfusion** | | | | | | | |  |
| No | 276 (79.3) | (Reference) |  | (Reference) |  | (Reference) |  |  |
| Yes | 72 (20.7) | 15,976.02 (7,553.99 – 24,398.04) | <0.001* | 26,540.54 (18,911.29 – 34,169.79) | <0.001* | 42,106.95 (23,653.30 – 60,560.59) | <0.001* |  |
| **Readmission** | | | | | | | |  |
| No | 298 (85.6) | (Reference) |  | (Reference) |  | (Reference) |  |  |
| Yes | 50 (14.4) | 6,482.68 (1,186.11 – 11,779.26) | 0.017 | 13,389.75 (4,264.96 – 22,514.55) | 0.004* | 18,282.81 (6,885.00 – 29,680.61) | 0.002* |  |
| Hospital cost is presented as USD and a value of inflated to 31 Dec 2019 based on end of fiscal quarter Australian Consumer Price index. Values are presented as estimated median (interquartile range) using bootstrapped quantile regression adjusted for CCI, preoperative anemia, emergency surgery, surgical technique, intraoperative fluid, and transfusion during admission. The estimated cost by intraoperative fluid corresponds with additional cost by each 1 L. *: P<0.016 | | | | | | | |  |

| S4 Table. Number of complications and hospital costs adjusted for predefined variables. Estimated median and 95% confidence intervals of variables at 25th, 50th, and 75th percentiles of hospital cost. | | | | | | | |
| --- | --- | --- | --- | --- | --- | --- | --- |
| **Variables** | **Number (proportion)** | **25th percentile** | | **50th percentile** | | **75th percentile** | |
|  |  | **Median (95%CI)** | **P value** | **Median (95%CI)** | **P value** | **Median (95%CI)** | **P value** |
| **Number of complications** | | | | | | | |
| No complication | 64 (18.4) | (Reference) |  | (Reference) |  | (Reference) |  |
| 1 complication | 59 (17) | -34.75 (-3,693.97 – 3,624.47) | 0.985 | 556.17 (-3,197.10 – 4,309.44) | 0.772 | 2,730.30 (-1,215.45 – 6,676.05) | 0.176 |
| 2 complications | 49 (14.1) | 1,415.98 (-2,018.66 – 4,850.62) | 0.42 | 1,669.22 (-1,968.92 – 5,307.35) | 0.369 | 1,789.08 (-4,257.26 – 7,835.43) | 0.563 |
| 3 complications | 56 (16.1) | 2,288.22 (-866.39 – 5,442.84) | 0.157 | 2,958.70 (-450.10 – 6,367.50) | 0.090 | 3,439.32 (-1,389.25 – 8,267.89) | 0.164 |
| 4 or more complications | 120 (34.5) | 9,924.32 (5,317.19 – 1,4531.44) | <0.001* | 14,502.49 (8,247.11 – 20,757.88) | <0.001* | 20,241.67 (4,804.11 – 35,679.23) | 0.011* |
| **CCI** | NA | 81.34 (-404.28 – 566.97) | 0.743 | 61.07 (-472.71 – 594.86) | 0.823 | 164.74 (-646.99 – 976.47) | 0.691 |
| **Anemia** | | | | | | | |
| No | 208 (59.8) | (Reference) |  | (Reference) |  | (Reference) |  |
| Yes | 140 (40.2) | 1,390.28 (-1,429.76 – 4,210.32) | 0.335 | 2,717.93 (-163.22 – 5,599.08) | 0.066 | 3,853 (-103 – 7,809.01) | 0.058 |
| **Emergency surgery** | | | | | | | |
| No | 97 (27.9) | (Reference) |  | (Reference) |  | (Reference) |  |
| Yes | 251 (72.1) | -812.01 (-3,559.68 – 1,935.66) | 0.563 | -2,443.24 (-5,510.33 – 623.85) | 0.120 | -4,731.20 (-8,867.13 – -595.27) | 0.026 |
| **Technique** | | | | | | | |
| Laparoscopy | 61 (17.5) | (Reference) |  | (Reference) |  | (Reference) |  |
| Laparotomy | 287 (82.5) | 523.06 (-2,223.87 – 3,270.00) | 0.709 | 2,082.78 (-975.38 – 5,140.94) | 0.183 | 3,822.35 (269.76 – 7,374.94) | 0.036 |
| **Intraoperative fluid** | NA | 1,606.02 (173.77 – 3,038.27) | 0.029 | 1,662.11 (-18.82 – 3,343.04) | 0.054 | 1,760.51 (-317.38 – 3,838.39) | 0.098 |
| **Transfusion** | | | | | | | |
| No | 276 (79.3) | (Reference) |  | (Reference) |  | (Reference) |  |
| Yes | 72 (20.7) | 10,086.28 (3,280.23 – 16,892.32) | 0.004* | 16,907.34 (5,317.68 – 28,497.00) | 0.005* | 31,317.77 (10,999.88 – 51,635.67) | 0.003* |
| **Readmission** | | | | | | | |
| No | 298 (85.6) | (Reference) |  | (Reference) |  | (Reference) |  |
| Yes | 50 (14.4) | 6,781.53 (2,543.81 – 11,019.25) | 0.002* | 10,476.72 (2,738.48 – 18,214.97) | 0.009* | 15,880.86 (5,410.91 – 26,350.81) | 0.003* |
| Hospital cost is presented as USD and a value of inflated to 31 Dec 2019 based on end of fiscal quarter Australian Consumer Price index. Values are presented as estimated median (interquartile range) using bootstrapped quantile regression adjusted for CCI, preoperative anemia, emergency surgery, surgical technique, intraoperative fluid, and transfusion during admission. The estimated cost by intraoperative fluid corresponds with additional cost by each 1 L. *: P<0.016. | | | | | | | |

| S5 Table. Severity of postoperative surgical complication and hospital costs adjusted for predefined variables. Estimated median and 95% confidence intervals of variables at 25th, 50th, and 75th percentiles of hospital cost. | | | | | | | |
| --- | --- | --- | --- | --- | --- | --- | --- |
| **Variables** | **Number (proportion)** | **25th percentile** | | **50th percentile** | | **75th percentile** | |
|  |  | **Median (95%CI)** | **P value** | **Median (95%CI)** | **P value** | **Median (95%CI)** | **P value** |
| **Severity of complications** | | | | | | | |
| CVD grade 0 | 64 (18.4) | (Reference) |  | (Reference) |  | (Reference) |  |
| CVD grade I | 59 (17.0) | 871.81 (-2,407.16 – 4,150.79) | 0.603 | 1,276.94 (-1,719.25 – 4,273.12) | 0.404 | 882.95 (-2,542.29 – 4,308.19) | 0.614 |
| CVD grade II | 137 (39.4) | 2,072.22 (-1,020.70 – 5,165.13) | 0.190 | 3,933.86 (938.10 – 6,929.62) | 0.011 | 6,777.20 (2,585.27 – 10,969.12) | 0.002* |
| CVD grade III | 21 (6.0) | 19,353.73 (6,627.39 – 32,080.08) | 0.003* | 21,073.98 (10,888.82 – 31,259.13) | <0.001* | 29,279.48 (14,632.23 – 43,926.72) | <0.001* |
| CVD grade IV | 44 (12.6) | 22,390.04 (9,826.74 – 34,953.34) | <0.001* | 36,073.14 (19,243.45 – 52,902.83) | <0.001* | 58,709.53 (35,478.72 – 81,940.34) | <0.001* |
| CVD grade V | 23 (6.6) | 10,905.49 (692.17 – 21,118.82) | 0.037 | 14,947.61 (3,820.09 – 26,075.13) | 0.009* | 17,548.77 (-12,059.80 – 47,157.34) | 0.247 |
| **CCI** | NA | -122.67 (-604.18 – 358.84) | 0.618 | 61.55 (-470.84 – 593.94) | 0.821 | -47.32 (-816.16 – 721.52) | 0.904 |
| **Anemia** | | | | | | | |
| No | 208 (59.8) | (Reference) |  | (Reference) |  | (Reference) |  |
| Yes | 140 (40.2) | 225.33 (-3,011.74 – 3,462.41) | 0.892 | 2,791.98 (-244.25 – 5,828.21) | 0.073 | 5,365.83 (1,588.50 – 9,143.16) | 0.006* |
| **Emergency surgery** | | | | | | | |
| No | 97 (27.9) | (Reference) |  | (Reference) |  | (Reference) |  |
| Yes | 251 (72.1) | -2,588.99 (-5,624.86 – 446.87) | 0.096 | -4,216.36 (-7,032.93 – -1,399.78) | 0.004* | -2,632.40 (-6,545.99 – 1,281.19) | 0.189 |
| **Technique** | | | | | | | |
| Laparoscopy | 61 (17.5) | (Reference) |  | (Reference) |  | (Reference) |  |
| Laparotomy | 287 (82.5) | 1,163.77 (-1,440.31 – 3,767.86) | 0.382 | 1,735.91 (-1,455.72 – 4,927.53) | 0.288 | 3,238.46 (-611.13 – 7,088.04) | 0.101 |
| **Intraoperative fluid** | NA | 971.47 (-521.00 – 2,463.94) | 0.203 | 1,683.80 (2.52 – 3,365.09) | 0.051 | 709.12 (-1,128.87 – 2,547.11) | 0.450 |
| **Transfusion** | | | | | | | |
| No | 276 (79.3) | (Reference) |  | (Reference) |  | (Reference) |  |
| Yes | 72 (20.7) | 11,299.76 (4,033.38 – 18,566.14) | 0.003* | 13,429.82 (6,124.90 – 20,734.75) | <0.001* | 12,993.93 (661.80 – 25,326.06) | 0.040 |
| **Readmission** | | | | | | | |
| No | 298 (85.6) | (Reference) |  | (Reference) |  | (Reference) |  |
| Yes | 50 (14.4) | 7,403.65 (2,578.49 – 12,228.82) | 0.003* | 6,832.28 (-2,270.21 – 15,934.78) | 0.143 | 13,434.05 (5,973.13 – 20,894.97) | <0.001* |
| Hospital cost is presented as USD and a value of inflated to 31 Dec 2019 based on end of fiscal quarter Australian Consumer Price index. Values are presented as estimated median (interquartile range) using bootstrapped quantile regression adjusted for CCI, preoperative anemia, emergency surgery, surgical technique, intraoperative fluid, and transfusion during admission. The estimated cost by intraoperative fluid corresponds with additional cost by each 1 L. *: P<0.016. | | | | | | | |

| S6 Table. Details of the hospital cost centres by the presence of complication. | | | | |
| --- | --- | --- | --- | --- |
| **Variables** | **NPC group (N=64)** | **PC group (N=284)** | **P value** | **Effect size** |
| Allied health | 147.35 (10.83 – 340.70), [3.23:1,558.67] | 655.06 (293.14 – 1,159.90), [0.00:13,141.91] | <0.001* | 0.34 |
| Anaesthesia/surgery | 6,301.10 (4,592.38 – 8,037.19), [0.00:19,105.00] | 6,352.59 (4,780.95 – 9,632.42), [0.00:80,479.34] | 0.454 | 0.04 |
| Blood products | 0.00 (0.00 – 0.00), [0.00:163.14] | 0.00 (0.00 – 240.62), [0.00:27,077.56] | <0.001* | 0.26 |
| ICU care | 0.00 (0.00 – 0.00), [0.00:2,578.41] | 0.00 (0.00 – 5,670.67), [0.00:134,595.44] | <0.001* | 0.31 |
| Medical | 939.75 (756.3 – 1,224.07), [219.25:3,489.96] | 1,822.92 (1,122.46 – 2,959.38), [119.38:36,839.49] | <0.001* | 0.39 |
| Medical emergency team call | 0.00 (0.00 – 0.00), [0.00:0.00] | 0.00 (0.00 – 0.00), [0.00:1,558.35] | 0.001* | 0.18 |
| Pathology/radiology | 445.22 (244.94 – 747.12), [0:1,459.73] | 1,045.33 (531.46 – 2,133.00), [0.00:19,524.79] | <0.001* | 0.36 |
| Ward | 3,293.70 (2,795.71 – 3,882.43), [105.65:13,535.20] | 5,869.61 (3,868.85 – 10,025.83), [51.32:167,555.59] | <0.001* | 0.39 |
| Readmission | 0.00 (0.00 – 0.00), [0.00:6,847.92] | 0.00 (0.00 – 0.00), [0.00:80,635.24] | 0.502 | 0.04 |
| Grand total | 11,663.56 (9,442.14 – 15,777.73), [5,182.17:32,547.51] | 19,805.41 (13,736.48 – 36,123.8), [5,724.96:46,0276.75] | <0.001* | 0.39 |
| Hospital cost is presented as USD and a value of inflated to 31 Dec 2019 based on end of fiscal quarter Australian Consumer Price index. Values are presented as median (interquartile range), [Min:Max]. NPC group: No postoperative complication group, PC group: Postoperative complication group, Effect size: common language effect size *r* for Mann-Whitney U test, *: Bonferroni corrected P value below 0.0056. | | | | |

| S7 Table. Details of the hospital cost centres classified by the number of complications. | | | | | | |
| --- | --- | --- | --- | --- | --- | --- |
| Variables | 1 Cx (N=59) | 2 Cx (N=49) | 3 Cx (N=56) | 4 or more Cx (N=120) | P value | Effect size |
| Allied health | 336.73 (20.13 – 649.88), [1.59:2,978.67] | 373.78 (221.00 – 1,132.47), [0.00:2,265.46] | 505.99 (198.61 – 1,099.17), [5.52:2,517.94] | 1,013.83 (552.10 – 1,393.47), [0.00:1,3141.91] | <0.001* | 0.23 |
| Anesthesia/surgery | 5,419.62 (4,284.30 – 7,810.72), [1,384.64:15,261.01] | 5,866.02 (4,419.90 – 7,796.92), [0.00:34,221.34] | 6,638.87 (4,846.67 – 8,370.93), [0.00:18,679.27] | 7,061.69 (4,955.47 – 12,406.36), [0.00:80,479.34] | 0.006 | 0.03 |
| Blood products | 0.00 (0.00 – 0.00), [0:1,407.35] | 0.00 (0.00 – 9.06), [0:5,154.39] | 0.00 (0.00 – 0.00), [0:4,364.03] | 83.76 (0.00 – 11,83.08), [0.00:27,077.56] | <0.001* | 0.19 |
| ICU care | 0.00 (0.00 – 760.87), [0.00:21,419.63] | 0.00 (0.00 – 1,256.15), [0.00:32,065.19] | 0.00 (0.00 – 872.72), [0.00:20,063.21] | 4,992.16 (0.00 – 14,665.87), [0.00:134,595.44] | <0.001* | 0.26 |
| Medical | 1,018.29 (621.70 – 1,484.62), [119.38:4,818.82] | 1,608.38 (1,040.53 – 2,249.94), [487.93:4,364.49] | 1,811.69 (1,099.90 – 2,316.65), [371.08:6,730.10] | 2,694.07 (1,692.40 – 4,399.27), [637.65:36,839.49] | <0.001* | 0.38 |
| Medical emergency team call | 0.00 (0.00 – 0.00), [0:155.13] | 0.00 (0.00 – 0.00), [0:170.62] | 0.00 (0.00 – 0.00), [0:509.03] | 0.00 (0.00 – 0.00), [0:1,558.35] | <0.001* | 0.05 |
| Pathology/radiology | 498.32 (205.82 – 730.79), [14.07:5306.05] | 725.03 (492.82 – 1,130.80), [0:2,862.93] | 1,022.39 (556.59 – 1,610.21), [72.08:3,173.06] | 2,128.98 (1,096.69 – 3,706.89), [170.51:19,524.79] | <0.001* | 0.40 |
| Ward | 3,296.24 (2,413.36 – 4,235.36), [51.32:19,008.56] | 4,675.64 (3,832.49 – 6,228.15), [1,569.04:11,495.67] | 5,990.94 (4,164.89 – 8,058.90), [119.81:19,140.8] | 9,667.69 (6,179.52 – 15,400.92), [138.57:167,555.59] | <0.001* | 0.46 |
| Readmission | 0.00 (0.00 – 0.00), [0.00:18,236.36] | 0.00 (0.00 – 0.00), [0.00:80,635.24] | 0.00 (0.00 – 0.00), [0.00:36,679.69] | 0.00 (0.00 – 0.00), [0.00:79,488.6] | 0.824 | 0.01 |
| Grand total | 12,788.65 (9,471.24 – 17,118.54), [5,724.96:6,5176.97] | 16,603.85 (13,179.38 – 20,930.34), [5,782.32:105,855.83] | 17,725.68 (13,445.19 – 24,117.8), [7,814.53:56,906.86] | 34,433.26 (22,464.34 – 61,801.16), [10,014.17:460,276.75] | <0.001* | 0.45 |
| Hospital cost is presented as USD and a value of inflated to 31 Dec 2019 based on end of fiscal quarter Australian Consumer Price index. Values are presented as median (interquartile range), [Min:Max]. Effect size: $\eta_{H}^{2}$ for Kruskal-Wallis H test, *: Bonferroni corrected P value below 0.0056. | | | | | | |

| S8 Table. Details of the hospital cost, classified by CVD. | | | | | | | | |
| --- | --- | --- | --- | --- | --- | --- | --- | --- |
| Variables | CVD1 (N=59) | CVD2 (N=137) | CVD3 (N=21) | CVD4 (N=44) | | CVD5 (N=23) | P value | Effect size |
| Allied health | 352.80 (18.39 – 650.51), [0.00:1,492.38] | 656.65 (295.50 – 1,138.88), [00.00:2,781.47] | 1,167.23 (420.36 – 1,312.86), [65.85:2,458.43] | | 1,156.86 (670.95 – 1,674.17), [5.50:13,141.91] | 352.40 (36.78 – 1,107.07), [1.59:2,184.55] | <0.001* | 0.21 |
| Anesthesia/surgery | 5,770.32 (4,643.19 – 7,657.81), [0.00:34,221.34] | 6,017.11 (4,511.12 – 8,341.02), [00.00:23,619.09] | 10,305.9 (6,341.43 – 14,188.59), [4,265.66:24,697.11] | | 8,194.11 (5,626.47 – 16,107.62), [1,851.68:80,479.34] | 6,340.4 (4,159.42 – 11,566.38), [3,155.19:14,741.17] | <0.001* | 0.06 |
| Blood products | 00.00 (00.00 – 00.00), [00.00:1,373.35] | 00.00 (00.00 – 139.53), [00.00:5,154.39] | 46.32 (00.00 – 404.25), [00.00:3,517.08] | | 677.88 (00.00 – 2,202.98), [00.00:27,077.56] | 95.76 (00.00 – 948.55), [00.00:6,562.70] | <0.001* | 0.21 |
| ICU care | 00.00 (00.00 – 00.00), [00.00:5,234.76] | 00.00 (00.00 – 1,494.88), [00.00:32,065.19] | ,1279.39 (00.00 – 7,194.70), [00.00:29,206.83] | | 18,553.68 (9,183.31 – 35,900.01), [00.00:134,595.44] | 7,704.40 (2,026.56 – 13,998.89), [00.00:7,3921.57] | <0.001* | 0.52 |
| Medical | 1,225.92 (810.51 – 1,651.76), [293.46:3,184.47] | 1,835.51 (1,268.70 – 2,564.94), [445.97:20,093.27] | 3,359.21 (2,111.89 – 4,398.43), [1,071.07:10,471.33] | | 4,000.29 (2,262.33 – 8,195.91), [487.93:36,839.49] | 756.57 (371.08 – 2,492.23), [119.38:5,636.92] | <0.001* | 0.35 |
| Medical emergency team call | 00.00 (00.00 – 00.00), [00.00:170.3] | 00.00 (00.00 – 00.00), [00.00:313.38] | 00.00 (00.00 – 00.00), [00.00:509.03] | | 00.00 (00.00 – 153.04), [00.00:875.05] | 00.00 (00.00 – 156.69), [00.00:1,558.35] | <0.001* | 0.09 |
| Pathology-Radiology | 586.82 (327.41 – 994.49), [16.36:2,458.78] | 977.98 (521.74 – 1,606.91), [14.07:4,337.04] | 2,862.93 (1,761.2 – 3,679.21), [00.00:5,859.34] | | 3,426.48 (1,685.34 – 5,382.79), [72.08:19,524.79] | 1,423.00 (498.32 – 3,341.15), [23.62:5,013.47] | <0.001* | 0.35 |
| Ward | 4,022.07 (3,295.98 – 5,706.20), [2,149.21:8,617.10] | 5,931.64 (4,138.07 – 9,227.09), [1,200.73:35,060.80] | 12,807.55 (7,753.41 – 19,162.04), [2,633.22:56,311.98] | | 11,644.10 (7,092.97 – 22,500.15), [2,283.61:167,555.59] | 1,569.04 (298.06 – 8,827.65), [51.32:18,063.71] | <0.001* | 0.37 |
| Readmission | 00.00 (00.00 – 00.00), [00.00:7,939.42] | 00.00 (00.00 – 00.00), [00.00:80,635.24] | 00.00 (00.00 – 1,522.68), [00.00:16,527.02] | | 00.00 (00.00 – 00.00), [00.00:45,815.35] | 00.00 (00.00 – 00.00), [00.00:00.00] | 0.081 | 0.02 |
| Grand total | 13,420.50 (10,562.15 – 16,902.17), [5,724.96:4,1051.24] | 18,653.20 (14,748.80 – 27,227.16), [6,087.08:105,855.83] | 38,871.61 (26,090.30 – 53,398.22), [16,797.46:99,866.35] | | 66,454.13 (31,882.87 – 9,3717.97), [5,782.32:460,276.75] | 21,815.72 (12,626.47 – 37,260.71), [7,058.36:92,747.48] | <0.001* | 0.42 |
| Hospital cost is presented as USD and a value of inflated to 31 Dec 2019 based on end of fiscal quarter Australian Consumer Price index. Values are presented as median (interquartile range), [Min:Max]. Effect size: $\eta_{H}^{2}$ for Kruskal-Wallis H test, *: Bonferroni corrected P value below 0.0056. | | | | | | | | |
